# Supplementary material for: Long-read sequencing unveils high-resolution HPV integration and its oncogenic progression in cervical cancer
Source: Nat Commun. 2022 May 10;13:2563. doi: 10.1038/s41467-022-30190-1 (PMC9091225; doi:10.1038/s41467-022-30190-1)

Long-read sequencing unveils high-resolution HPV integration and its oncogenic progression in cervical cancer

Liyuan Zhou, Qiongzi Qiu, Qing Zhou, Jianwei Li, Mengqian Yu, Kezhen Li, Lingling Xu, Xiaohui Ke, Haiming Xu, Bingjian Lu, Hui Wang, Weiguo Lu, Pengyuan Liu, Yan Lu

Supplementary Data

Table S1. Quality metrics of ONT long read sequencing libraries.

Table S2. Genes potentially disrupted by HPV integration breakpoints.

Table S3. Integration breakpoints and their involved events.

Table S4. Details of PCR validation of breakpoints in clonal integration events.

Table S5. Quality metrics of RNA sequencing libraries.

Table S6. Statistical test and its associated significance of comparison between intSample and intRegion for each feature.

Table S7. List of PCR primers and siRNAs used in this study.

Figure S1. PCR validation of breakpoints in clonal integration events.

Figure S2. The base coverage of regions around several integration events in the human genome.

Figure S3. PCR validation of the ECC DNA structure

Figure S4. GO and KEGG enrichment analysis of genes potentially disrupted by HPV integration.

Figure S5. Expression level of genes around integration breakpoints and on affected genomic regions resulting from clonal integration events.

Figure S6. Knockdown assays of LENG9 in cervical cancer cell line CaSki.

Figure S7. Comparative expression level of LINC00290 and HPV E6/E7 genes in 103 cervical tumors.

Figure S8. Structural variation landscape in cervical cancer.

Figure S9. Genomic properties of structural variations in different regions.

Figure S10. Seven additional clonal integration events with one supporting chimeric read in ZLR-11.

Table S1 Quality metrics of ONT long read sequencing data

Patient-ID	Reads	Sequences (Gb)	N50Len	N90Len	MeanLen	MaxLen	MeanQual	Mapping Rate
ZLR-01	14,147,490	103.6	8575	4846	7322	184336	8.6	0.968
ZLR-02	9,549,474	87.7	13840	4994	9187	301188	9.6	0.958
ZLR-03	15,726,406	85.0	9892	2350	5403	145349	9.3	0.948
ZLR-04	16,886,600	96.8	9936	2589	5731	137249	9.2	0.945
ZLR-05	11,072,865	92.7	11976	4821	8367	122305	9.0	0.959
ZLR-06	18,183,696	120.9	10830	3090	6646	144644	9.1	0.959
ZLR-07	10,072,705	90.3	12096	5188	8962	152051	8.8	0.964
ZLR-08	19,284,204	119.6	8891	3229	6201	696229	11.2	0.956
ZLR-09	25,981,598	105.5	7803	1656	4059	129929	9.1	0.950
ZLR-10	11,695,389	89.2	10720	4042	7631	127396	9.5	0.967
ZLR-11	13,144,570	95.8	11282	3690	7290	136647	8.9	0.960
ZLR-12	10,564,581	87.3	13115	4072	8265	140681	9.0	0.961
ZLR-13	13,690,031	111.1	11528	4403	8111	121077	9.3	0.963
ZLR-14	10,926,167	102.7	13122	5202	9398	135722	9.2	0.964
ZLR-15	15,439,607	116.2	10665	4426	7523	154834	9.6	0.962
ZLR-16	16,596,177	129.7	10726	4607	7812	136714	9.4	0.963

Table S2 Genes potentially disrupted by integration breakpoints

Patient-ID	Human					HPV		
	Chr	Pos	Gene	Region		HPV Type	Pos	Gene
ZLR-01	chr4	182138890	LINC00290;TEMN3-AS1	intergenic		16	3368	E2,E4
ZLR-01	chr4	182273142	LINC00290;TEMN3-AS1	intergenic		16	5305	L2
ZLR-02	chr11	64975398	CAPN1	intronic		16	2138	E1
ZLR-02	chr11	64974748	CAPN1	intronic		16	3570	E2,E4
ZLR-03	chr10	104080707	GBF1	intronic		16	2191	E1
ZLR-03	chr10	104084259	GBF1	intronic		16	6139	L1
ZLR-03	chr10	104080663	GBF1	intronic		16	2186	E1
ZLR-04	chr20	14754684	MACROD2	intronic		16	2991	E2
ZLR-04	chr20	14842487	MACROD2	intronic		16	2834	E2
ZLR-04	chr20	14840951	MACROD2	intronic		16	3086	E2
ZLR-06	chr3	137186017	IL20RB;SOX14	intergenic		16	6291	L1
ZLR-06	chr4	81210954	FGF5	UTR3		16	2026	E1
ZLR-06	chr4	81212477	FGF5	downstream		16	1825	E1
ZLR-06	chr9	75392601	TMC1	intronic		16	1093	E1
ZLR-06	chr9	75532466	ALDH1A1	intronic		16	1451	E1
ZLR-06	chr9	75413449	TMC1	intronic		16	1833	E1
ZLR-06	chr3	137188183	IL20RB;SOX14	intergenic		16	1775	E1
ZLR-06	chr8	49845734	SNAI2;C8orf22	intergenic		16	306	E6
ZLR-06	chr12	103589765	LOC101929058;C12orf42	intergenic		16	7339	URR
ZLR-06	chr8	49845725	SNAI2;C8orf22	intergenic		16	7050	L1
ZLR-06	chr1	48168486	FOXD2;TRABD2B	intergenic		16	2339	E1
ZLR-06	chr12	103589678	LOC101929058;C12orf42	intergenic		16	3478	E2,E4
ZLR-06	chr9	75433908	TMC1	intronic		16	4628	L2
ZLR-07	chr2	146386905	TEX41;PABPC1P2	intergenic		16	1955	E1
ZLR-07	chr2	146498465	TEX41;PABPC1P2	intergenic		16	909	E1
ZLR-08	chr19	54983022	CDC42EP5	intronic		16	2274	E1
ZLR-08	chr19	54974840	LENG9	UTR5		16	2636	E1
ZLR-08	chr19	54970568	LENG8	intronic		16	7029	L1
ZLR-08	chr19	54974755	LENG9	exonic		16	7906	URR
ZLR-08	chr19	54984924	CDC42EP5	upstream		16	2024	E1
ZLR-08	chr19	54978308	CDC42EP5	intronic		16	3050	E2
ZLR-08	chr19	54970599	LENG8	intronic		16	1686	E1
ZLR-08	chr19	54983028	CDC42EP5	intronic		16	2276	E1
ZLR-08	chr19	54974755	LENG9	exonic		16	131	E6
ZLR-08	chr19	54974756	LENG9	exonic		16	6486	L1
ZLR-08	chr19	54979846	CDC42EP5	intronic		16	4968	L2
ZLR-08	chr19	54978038	CDC42EP5	intronic		16	1642	E1
ZLR-09	chr4	142864411	IL15;INPP4B	intergenic		16	4003	E5
ZLR-09	chr4	142649381	IL15	intronic		16	3226	E2
ZLR-10	chr10	79694897	DLG5-AS1;POLR3A	intergenic		16	3411	E2,E4
ZLR-11	chr15	86031825	AKAP13	intronic		16	2797	E2,E1
ZLR-11	chr8	128323818	CASC21;CASC8	ncRNA_intronic		16	2799	E2,E1
ZLR-11	chr8	128323153	CASC21;CASC8	ncRNA_intronic		16	4464	L2
ZLR-11	chr8	128323315	CASC21;CASC8	ncRNA_intronic		16	478	E6
ZLR-11	chr8	128419658	CASC8	ncRNA_intronic		16	2636	E1
ZLR-11	chr15	85988611	AKAP13	intronic		16	4774	L2
ZLR-11	chr8	128419658	CASC8	ncRNA_intronic		16	7784	URR
ZLR-11	chr8	128323084	CASC21;CASC8	ncRNA_intronic		16	4368	L2
ZLR-11	chr8	128419610	CASC8	ncRNA_intronic		16	7781	URR
ZLR-11	chr15	86014813	AKAP13	intronic		16	5226	L2
ZLR-12	chr17	36095944	HNF1B	intronic		16	3409	E2,E4
ZLR-12	chr17	36095602	HNF1B	intronic		16	37	URR
ZLR-12	chr17	36095602	HNF1B	intronic		16	5979	L1
ZLR-12	chr17	36094082	HNF1B	intronic		16	4573	L2
ZLR-13	chr13	73982893	KLF5;LINC00392	intergenic		16	3331	E2
ZLR-13	chr13	73982772	KLF5;LINC00392	intergenic		16	4686	L2
ZLR-13	chr13	73982955	KLF5;LINC00392	intergenic		16	3285	E2
ZLR-14	chr13	73922258	KLF5;LINC00392	intergenic		16	2728	E1
ZLR-14	chr13	73955322	KLF5;LINC00392	intergenic		16	1478	E1
ZLR-15	chr14	91451921	RPS6KA5	intronic		16	2972	E2
ZLR-15	chr14	91451918	RPS6KA5	intronic		16	3049	E2
ZLR-16	chr17	57757805	CLTC	intronic		58	3821	E2
ZLR-16	chr17	57879163	VMP1	intronic		58	6409	L1

Table S3 Integration breakpoints and their related events.

Patient-ID	Breakpoints	Events involved	GT:DR:DV
ZLR-01	Chr4:182138890_HPV16:3368	e	0/0:22:7
ZLR-01	Chr4:182273142_HPV16:5305	e	0/0:15:5
ZLR-02	Chr11:64974748_HPV16:3570	e	0/1:8:14
ZLR-02	Chr11:64975398_HPV16:2138	e	1/1:1:18
ZLR-03	Chr10:104080707_HPV16:2191	e	0/1:20:45
ZLR-03	Chr10:104084259_HPV16:6139	e	1/1:8:54
ZLR-03	Chr10:104080663_HPV16:2186	@	0/0:20:4
ZLR-04	Chr20:14754684_HPV16:2991	e	0/1:13:23
ZLR-04	Chr20:14840951_HPV16:3086	e	0/1:4:16
ZLR-04	Chr20:14842487_HPV16:2834	@	1/1:0:3
ZLR-06	Chr1:48168486_HPV16:2339	@	0/1:12:8
ZLR-06	Chr3:137186017_HPV16:6291	e1	0/0:34:12
ZLR-06	Chr3:137188183_HPV16:1775	e1	1/1:3:19
ZLR-06	Chr4:81210954_HPV16:2026	@	0/0:11:4
ZLR-06	Chr4:81212477_HPV16:1825	@	1/1:0:4
ZLR-06	Chr8:49845725_HPV16:7050	@	0/1:19:31
ZLR-06	Chr8:49845734_HPV16:306	@	1/1:0:25
ZLR-06	Chr9:75392601_HPV16:1093	@	0/1:12:16
ZLR-06	Chr9:75413449_HPV16:1833	e2	0/1:29:39
ZLR-06	Chr9:75433908_HPV16:4628	@	0/0:35:3
ZLR-06	Chr9:75532466_HPV16:1451	e2	0/1:14:33
ZLR-06	Chr12:103589678_HPV16:3478	@	0/1:7:4
ZLR-06	Chr12:103589765_HPV16:7339	@	1/1:0:5
ZLR-07	Chr2:146386905_HPV16:1955	@	0/0:9:3
ZLR-07	Chr2:146498465_HPV16:909	@	0/0:29:3
ZLR-08	Chr19:54983022_HPV16:2274	@	1/1:1:22
ZLR-08	Chr19:54974840_HPV16:2636	e2	1/1:0:45
ZLR-08	Chr19:54970568_HPV16:7029	e1	0/1:18:19
ZLR-08	Chr19:54974755_HPV16:7906	@	0/0:19:3
ZLR-08	Chr19:54984924_HPV16:2024	@	0/1:4:15
ZLR-08	Chr19:54978308_HPV16:3050	e3	1/1:0:5
ZLR-08	Chr19:54970599_HPV16:1686	e4	1/1:0:18
ZLR-08	Chr19:54983028_HPV16:2276	e1/e2/e3/e4/e5	1/1:0:84
ZLR-08	Chr19:54974755_HPV16:131	@	0/0:19:3
ZLR-08	Chr19:54974756_HPV16:6486	@	1/1:0:4
ZLR-08	Chr19:54979846_HPV16:4968	@	1/1:0:4
ZLR-08	Chr19:54978038_HPV16:1642	e5	1/1:0:4
ZLR-09	Chr4:142649381_HPV16:3226	@	0/1:18:19
ZLR-09	Chr4:142864411_HPV16:4003	@	0/1:9:20
ZLR-10	Chr10:79694897_HPV16:3411	@	0/0:16:6
ZLR-11	Chr8:128323084_HPV16:4368	e1/e3/e4	1/1:2:268
ZLR-11	Chr8:128323818_HPV16:2799	@	1/1:0:18
ZLR-11	Chr8:128323315_HPV16:478	e3	0/1:8:13
ZLR-11	Chr8:128419658_HPV16:7784	e1/e2/e3	1/1:0:266
ZLR-11	Chr8:128419658_HPV16:2636	@	1/1:0:8
ZLR-11	Chr15:85988611_HPV16:4774	e2/e3	1/1:1:52
ZLR-11	Chr15:86014813_HPV16:5226	@	0/0:22:5
ZLR-11	Chr15:86031825_HPV16:2797	e4	1/1:17:74
ZLR-11	Chr8:128323153_HPV16:4464	@	1/1:0:12
ZLR-11	Chr8:128419610_HPV16:7781	@	1/1:0:7
ZLR-12	Chr17:36094082_HPV16:4573	e3	1/1:4:18
ZLR-12	Chr17:36095602_HPV16:5979	e1/e2/e3	1/1:1:58
ZLR-12	Chr17:36095602_HPV16:37	@	0/1:1:4
ZLR-12	Chr17:36095944_HPV16:3409	e1/e2	1/1:1:44
ZLR-13	Chr13:73982772_HPV16:4686	@	1/1:0:4
ZLR-13	Chr13:73982893_HPV16:3331	e	1/1:0:154
ZLR-13	Chr13:73982955_HPV16:3285	e	1/1:0:8
ZLR-14	Chr13:73922258_HPV16:2728	e	0/1:18:24
ZLR-14	Chr13:73955322_HPV16:1478	e	1/1:5:23
ZLR-15	Chr14:91451918_HPV16:3049	e	1/1:0:54
ZLR-15	Chr14:91451921_HPV16:2972	e	1/1:0:55
ZLR-16	Chr17:57757805_HPV58:3821	e	0/0:21:4
ZLR-16	Chr17:57879163_HPV58:6409	e	0/1:20:12

Note: "@" indicates that breakpoints are not involved in any detected clonal integration events; "e" indicates that breakpoints are involved in that clonal integration **e**vent; GT: genotype; DR: number of high-quality reference reads; DV: number of high-quality variant reads.

Table S4. Details of PCR validation of breakpoints in clonal integration events

ID	Patient-ID	Breakpoints	Forward primer	Reverse primer	Expected PCR product size (bp)	Validation
1	ZLR-01	Chr4:182273124_HPV16:5305	GCCCGAATATGATGAGGCAC	ACCTAATGGCTGACCACGAC	959bp	YES
2	ZLR-01	HPV16:3374_Chr4:182138890	TGCCAACACTGGCTGTATCAA	AGCCACTTACTGAGCATTCCC	978bp	YES
3	ZLR-02	Chr11:64974748_HPV16:3572	AGCCACTTACTGAGCATTCCC	CATGCGGGTGGTCAGGTAAT	735bp	YES
4	ZLR-02	HPV16:4051_HPV16:6654	TAGCCGATGCACGTTTTGTG	GTGGTGGGTGTAGCTTTTCG	687bp	YES
5	ZLR-02	HPV16:2144_Chr11:64975399	TGTATGGAGACACGCCAGAA	CGACTCATGCAGACCAAGGG	730bp	YES
6	ZLR-08	HPV16:1686_Chr19:54970602	GGGTGGTTGCAGTCAGTACA	CAGTCCCTCCCTAGCTCCAA	598bp	YES
7	ZLR-08	HPV16:1691_Chr19:54978038	CAACGTGTTGCGATTGGTGT	AGGTTGTAGTCTCAAGATCAGGT	410bp	YES
8	ZLR-12	Chr11:100590122_HPV16:2599	TGTCAGGCACAGCTAAACGA	AGCACATTCTAGGCGCATGT	520bp	NO
9	ZLR-12	HPV16:4572_Chr17:36094082	CGCTGCTTTTGTCTGTGTCT	AGAGGTGGGGAGTGCTATCT	810bp	YES
10	ZLR-12	Chr17:36095594_HPV16:5983	GCTACCACCTCCCTTTGTGA	ATTTGCAGTAGACCCAGAGCC	967bp	YES
11	ZLR-13	Chr13:73989111_Chr13:73980114	AGGCCACTGACTCCAGATTT	TGCTACTCCTGGCCCTATCT	463bp	YES
12	ZLR-11	HPV16:473_Chr8: 128323314	GCGACCCAGAAAGTTACCAC	AGCAGTGATCCGTTTATCCCC	519bp	YES
13	ZLR-11	Chr15:86031825_HPV16:2797	AGGTTTTTGGTTAGGGAACAGTTG	ATGGTGCAGCTAACACAGGT	687bp	YES
14	ZLR-16	Chr17:57757805_HPV58:5597	TAGAACTGCAAATGAGATGGGGT	TCGTACTCGCAGTGGAAAGC	577bp	YES
15	ZLR-16	HPV58:4600_HPV58:7602	CAGGAGCAGGCGATATGTCA	CACCATCCACCAAACGCAAA	974bp	YES
16	ZLR-16	HPV58:6714_HPV58:3820	TGTAAACTGGCAGACGGAGG	GTACACAGGGGACAAAGCGA	574bp	YES
17	ZLR-16	HPV58:6407_Chr17:57879164	CACGCTGTAGCCAATAAGGC	AAACCAATGCAATGTTACCGAAGA	937bp	YES

Note: Only the breakpoints involved in the clonal integration event with less than three supporting chimeric reads were confirmed by PCR and Sanger sequencing.

Table S5. Quality metrics of RNA sequencing libraries.

Samples	Reads	Mean Quality Score	% of >=30 Bases	Mapping rate (%)
ZLR-01	60,569,930	35.2	88.8	87.3
ZLR-02	63,322,460	35.3	89.3	90.2
ZLR-03	67,710,800	35.3	89.5	90.1
ZLR-04	81,848,382	36.0	92.1	92.9
ZLR-05	58,941,818	37.5	95.9	92.9
ZLR-06	167,903,836	36.5	92.4	88.8
ZLR-07	132,446,838	36.9	93.6	90.4
ZLR-08	120,230,506	36.6	92.8	89.5
ZLR-09	101,007,676	37.0	93.2	91.2
ZLR-10	95,936,494	37.7	95.0	92.9
ZLR-11	95,951,638	36.6	92.8	89.5
ZLR-12	137,763,726	37.5	95.2	91.6
ZLR-13	42,200,030	37.4	94.9	92.3
ZLR-14	135,594,710	39.7	98.6	94.6
ZLR-15	110,793,764	37.2	93.7	91.4
ZLR-16	119,187,674	39.0	97.7	73.0

Table S6. Statistical test and its associated significance of comparison between intSample and intRegion for each feature.

SV type	Feature	Statistical test type	FDR of comparison between intSample and intRegion
DEL	SV length	Wilcoxon test	0.3877
DUP	SV length	Wilcoxon test	0.0101
INS	SV length	Wilcoxon test	0.8425
INV	SV length	Wilcoxon test	0.4735
INVDUP	SV length	Wilcoxon test	0.1081
BND	Normalized distance to integration site	Kolmogorov–Smirnov test	0.9071
DEL	Normalized distance to integration site	Kolmogorov–Smirnov test	0.4069
DUP	Normalized distance to integration site	Kolmogorov–Smirnov test	1.54E-06
INS	Normalized distance to integration site	Kolmogorov–Smirnov test	1
INV	Normalized distance to integration site	Kolmogorov–Smirnov test	0.3159
INVDUP	Normalized distance to integration site	Kolmogorov–Smirnov test	0.0034
BND	Replication timing	Kolmogorov–Smirnov test	0.3253
DEL	Replication timing	Kolmogorov–Smirnov test	0.0107
DUP	Replication timing	Kolmogorov–Smirnov test	0.0001
INS	Replication timing	Kolmogorov–Smirnov test	0.2380
INV	Replication timing	Kolmogorov–Smirnov test	0.4465
INVDUP	Replication timing	Kolmogorov–Smirnov test	0.0025
BND	Normalized distance to TAD	Kolmogorov–Smirnov test	0.2380
DEL	Normalized distance to TAD	Kolmogorov–Smirnov test	0.7724
DUP	Normalized distance to TAD	Kolmogorov–Smirnov test	0.0025
INS	Normalized distance to TAD	Kolmogorov–Smirnov test	0.8425
INV	Normalized distance to TAD	Kolmogorov–Smirnov test	0.4418
INVDUP	Normalized distance to TAD	Kolmogorov–Smirnov test	1.02E-05
DUP	pLI score	Wilcoxon test	0.1306
INS	pLI score	Wilcoxon test	0.8425
INV	pLI score	Wilcoxon test	0.9071
INVDUP	pLI score	Wilcoxon test	0.5871
BND	z-score	Chi-squared test	0.4835
DEL	z-score	Chi-squared test	0.5871
DUP	z-score	Chi-squared test	0.3356
INV	z-score	Chi-squared test	0.9071
INVDUP	z-score	Chi-squared test	1

Table S7. List of PCR primers and siRNAs used in this study

Ptimers	Sequences	Size	Comments
E2-F	TGGAAACACATGCGCCTAGA	612bp	
E2-R	CGTCTGTGTTTCTTCGGTGC		
L2-F	ACTTGCTCCTGTAAGACCCC	640bp	
L2-R	ACGCCTAGAGGTTAATGCTGG		
H1-F	GTGAGGAGAGGACAGCATAACC	214bp	
H1-R	AGGTGGGACTAGGGCATCTT		
H2-F	GCTGTATTTGTTCCTCCCAGC	566bp	
H2-R	GACCAGTTTGTTGTCTTGCGA		
J1-F	GACCCTGGTATGGGTGTGC	607bp	Junction 1
J1-R	AGGGGATAAACGGATCACTGC		
J2-F	GGGTTCTGTGCCATGAATGC	544bp	Junction 2
J2-R	TGTACGTTTCCTGCTTGCCA		
H3-F	GTTGTACCAAAAGCTGCGCC	376bp	
H3-R	ATTTCCCCCAAGGCAAGCTA		
H4-F	TTTTCTGCCCTCTGCTCCAC	375bp	
H4-R	TGGCTCTGCGAAGAATTCCA		
COX5B-F	GGGCACCATTTTCCTTGATCAT	119bp	
COX5B-R	AGTCGCCTGCTCTTCATCAG		
pBR322-F	GACTATCGTCGCCGCACTTA	179bp	
pBR322-R	GACCAGTGACGAAGGCTTGA		
pUC19-F	TGTAAAACGACGGCCAGTGA	137bp	
pUC19-R	TGTGTGGAATTGTGAGCGGA		
pUG72-F	CCCTTGGCGAAAAGTCCTCT	106bp	
pUG72-R	TAGACGACAAAGGCGATGCA		
siRNA_1	CTGCTGATTTGGGAACACT	/	siRNAs for LENG9 gene
siRNA_2	GGCTGAGCTTTAGAAAGCT	/	
siRNA_3	GCTGAGTACACTACAGTCT	/	
qPCR_F	GCCCGCCTTAGTGTTGCA	/	qPCR primers for LENG9 gene
qPCR_R	GCCAGGGTCAGGTGTAGGTTC	/	

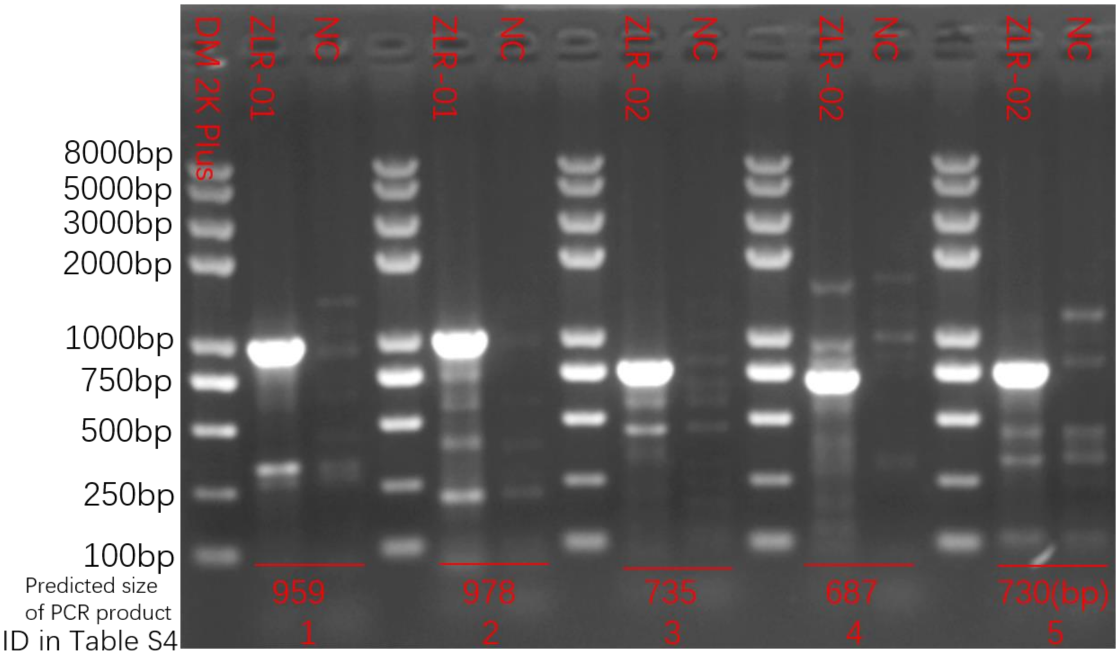

Figure S1. PCR validation of breakpoints in clonal integration events. Representative validation results of integration events are shown. Two independent experiments give the similar results. IDs of samples with detected integration events are indicated at the top. NC is a randomly selected sample without integration in the corresponding breakpoint. Two rows of numbers at the bottom are the predicted PCR product size and ID (the first column) in Table S4.

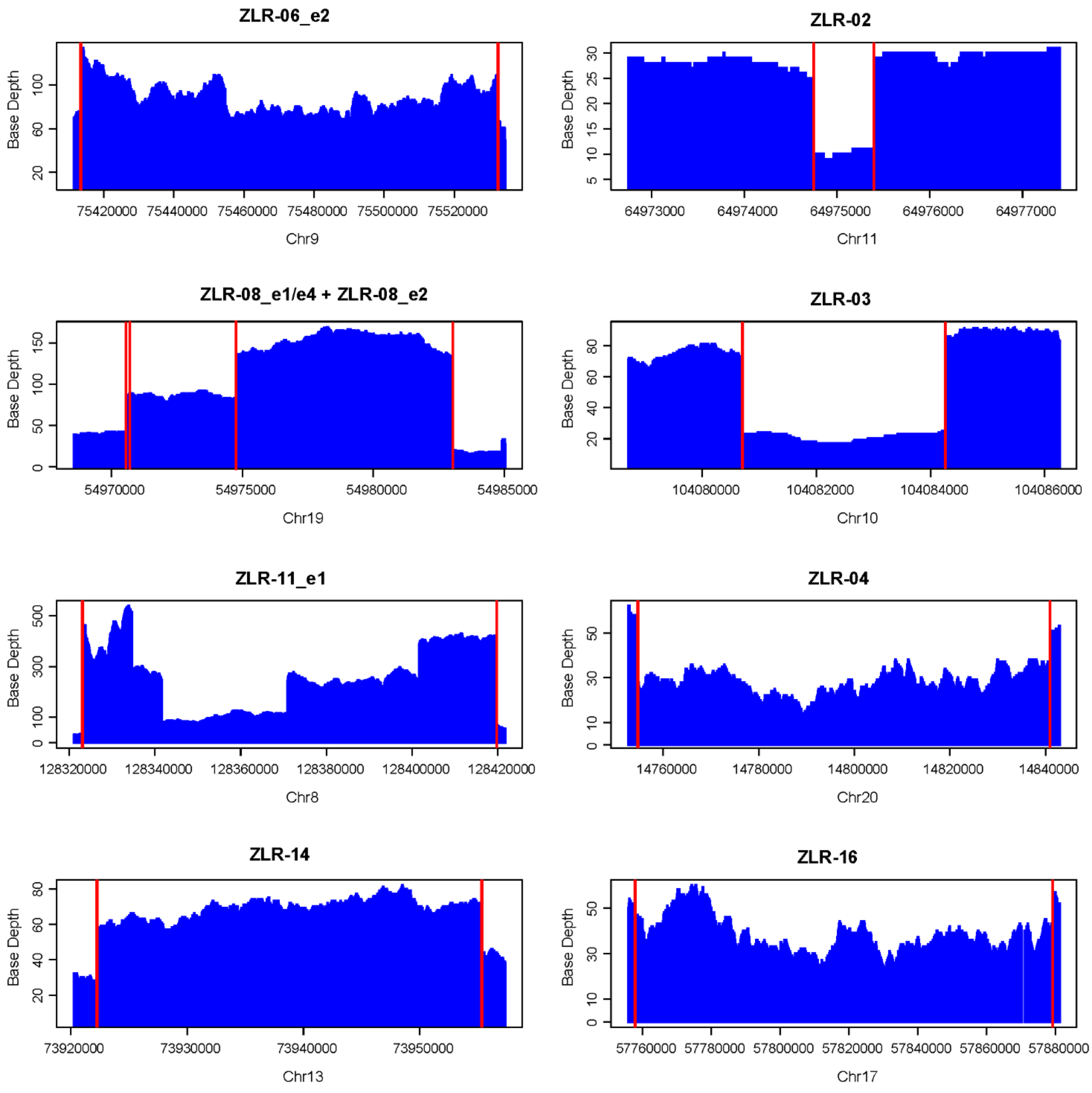

Figure S2. The base coverage of regions around several integration events in the human genome. Blue histograms show the depth of target regions (y-axis), with the indicated chromosomes and positions (x-axis). The red lines indicate the HPV integration breakpoint positions. For comparison, four types of ECC-related deletion in clonal integration events are shown in the left panel; four types of normal deletion in clonal integration events are shown in the right panel.

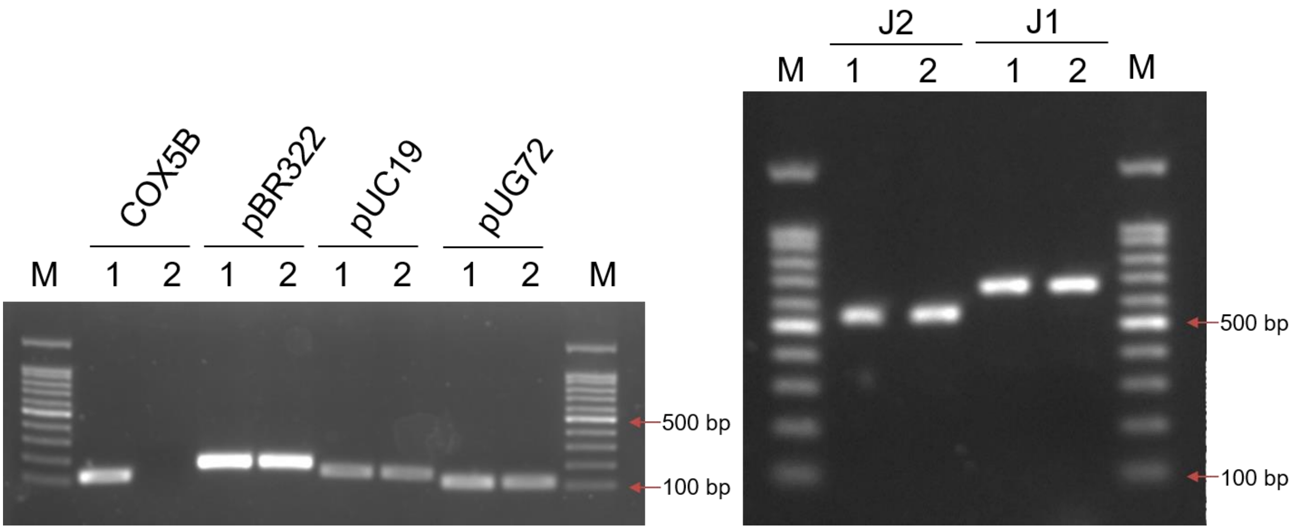

Figure S3. PCR validation of the ECC DNA structure. M: Marker; Lane 1: PCR with genomic DNA extracted from ZLR-11 as template; Lane 2: PCR with genomic DNA after the linear DNA removal as template; COX5B was a negative control for PCR reaction with linear DNA template; three internal plasmids, pBR322, pUC19 and pUG72, were positive controls for PCR reaction with circular DNA template. J2 and J1 were virus-human junction sites in ZLR-11_e1. Two independent experiments give the similar results.

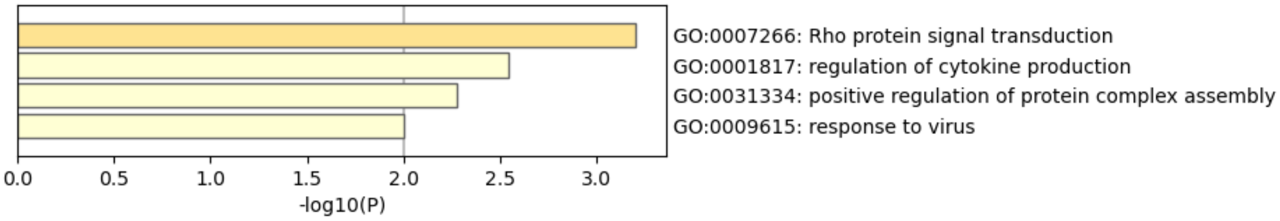

Figure S4. GO and KEGG enrichment analysis of genes potentially disrupted by HPV integration. Combined list of genes nearby (about 500 kb) integration breakpoints and on the affected genomic regions by clonal integration events were submitted to Metascape, a web portal for GO and KEGG enrichment analysis (https://metascape.org/gp/index.html).

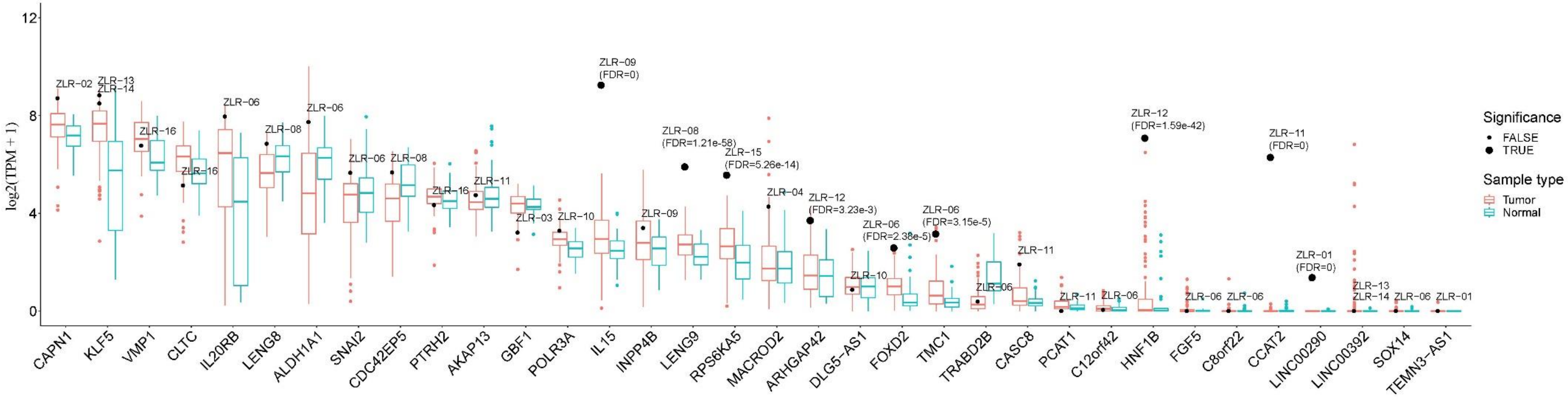

Figure S5. Expression level of genes around integration breakpoints and on affected genomic regions resulting from clonal integration events. The overall expression level in all tumors (n=103) and adjacent normal samples (n=39) is shown by boxplot. The expression level of a gene in an integrated sample is highlighted by black dots. Two-tailed p-values for changes in gene expression were generated by probability of the z-score occurring within a standard normal distribution and adjusted by the false discover rate (FDR). Significantly up-regulated genes with FDR < 0.05 are highlighted by dots of larger size, and numbers in parentheses indicate actual p-values. Thirty-four of 38 genes have available expression data. Data are shown in boxplots. The thick line in the box is median, and the box spans from Q1 (25th percentile) to Q3 (75th percentile). The whiskers extend to the most extreme observation within 1.5 times the interquartile range (IQR=Q3–Q1) from the nearest quartile. Outliers are represented by circles beyond the whiskers. Source data are provided as a Source Data file.

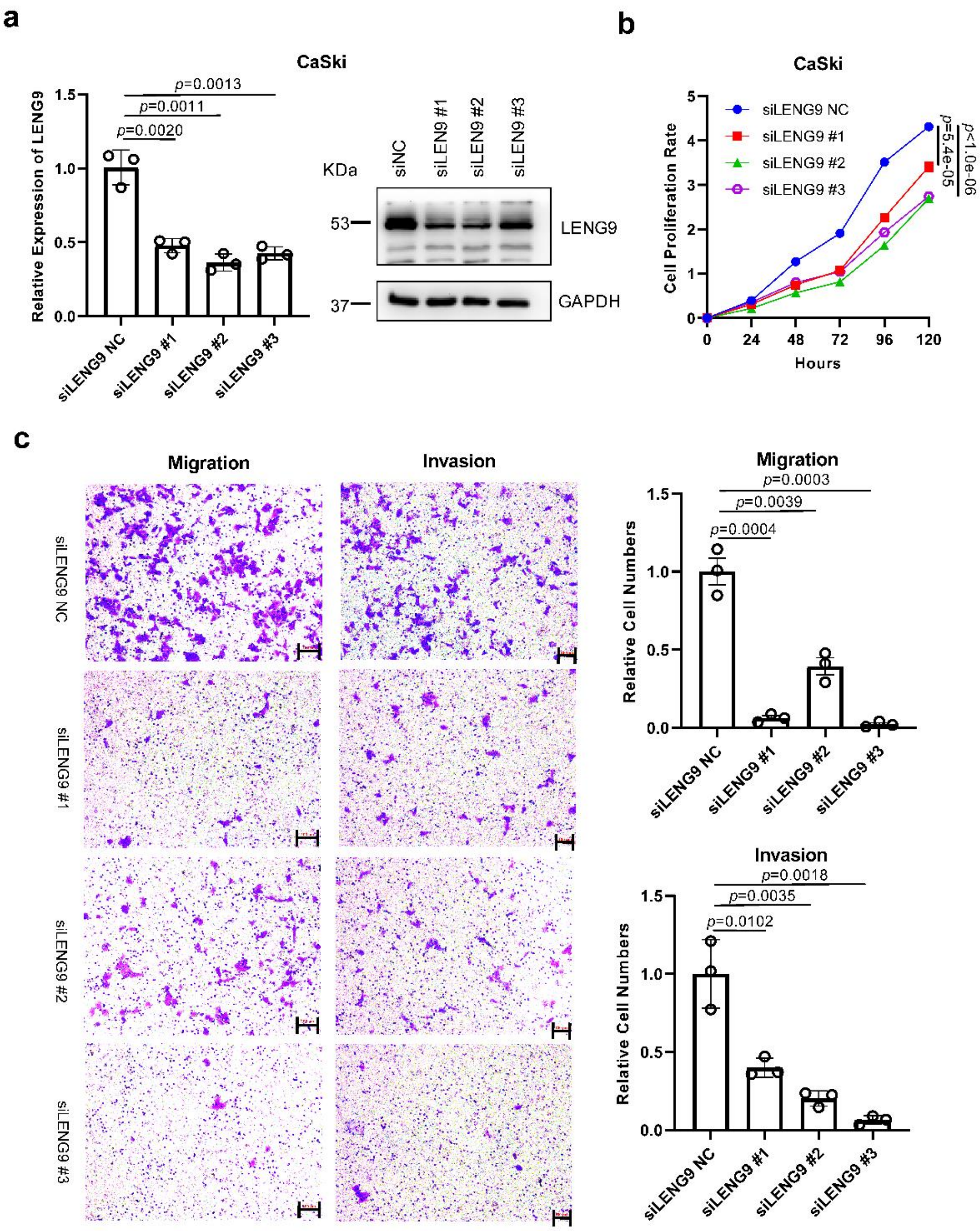

Figure S6. Knockdown assays of LENG9 in cervical cancer cell line CaSki. (a) The expression of LENG9 in CaSki cells transfected with three siRNAs targeting LENG9 and siRNA negative control. The expression levels of LENG9 were evaluated by both qRT-PCR and western blot. Data are presented as mean values +/- SEM (n=3) (**b**) Cell proliferation in CaSki cells transfected with three siRNAs targeting LENG9 and siRNA negative control, assessed by CCK8 (n=5). (**c**) Cell migration and invasion in CaSki cells transfected with three siRNAs targeting LENG9 and siRNA negative control, assessed by transwell assays. Scale bar, 100μm. Quantification of migration and invasion cells was summarized as histograms. Data are presented as mean values +/- SEM (n=3). Two sample t test was used for comparing the difference between siRNA and NC groups. Source data are provided as a Source Data file.

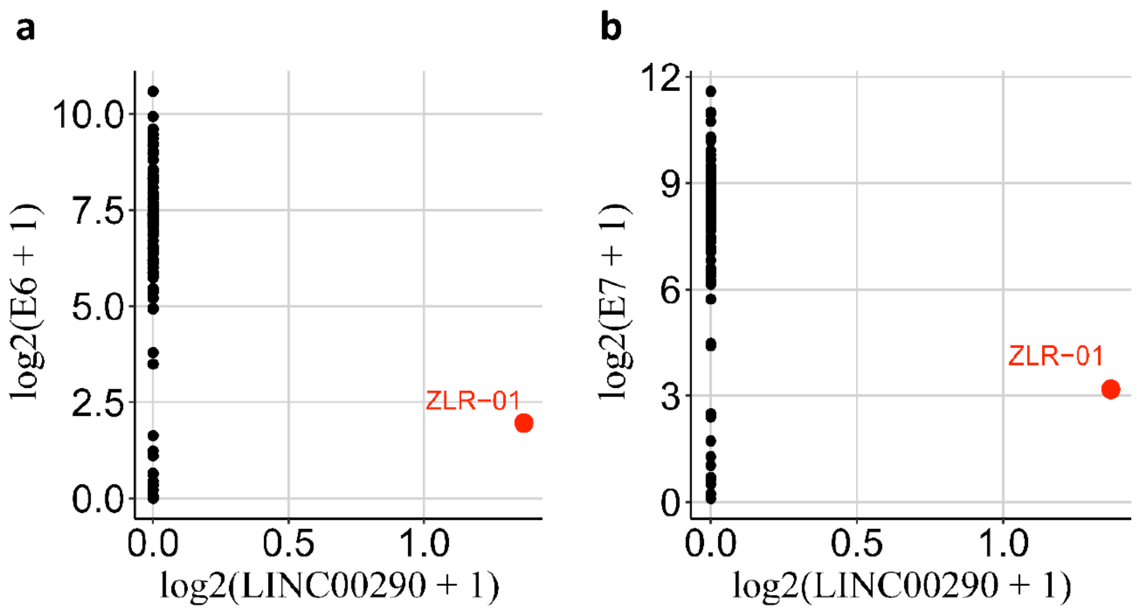

Figure S7. Comparative expression level of LINC00290 and HPV E6/E7 genes in 103 cervical tumors. (**a**) E6. (**b**) E7. Source data are provided as a Source Data file.

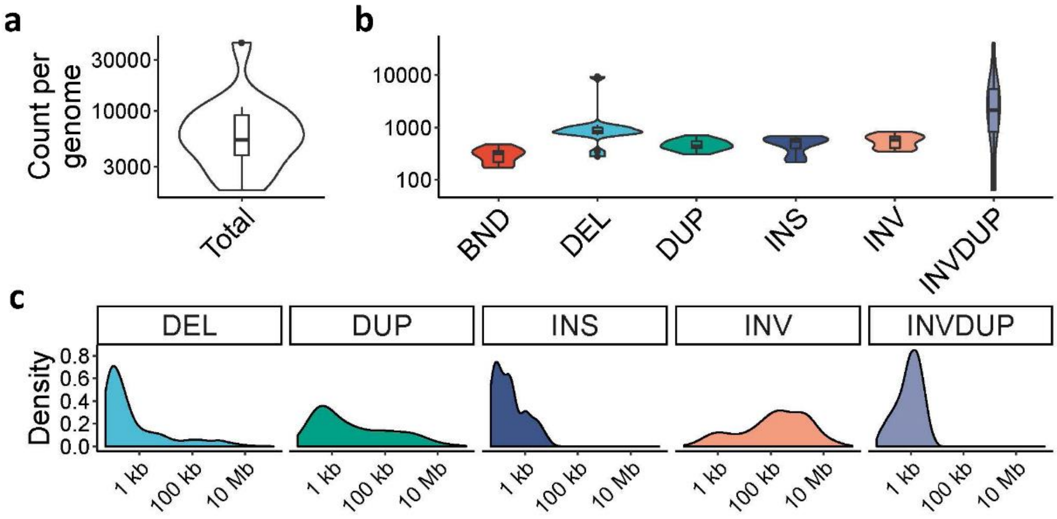

Figure S8. Structural variation landscape in cervical cancer. (**a-b**) SV count per genome (n=15). (**c**) Size distribution of five SV types. In panels a and b, the data are shown in violin plot with the addition of kernel density plot including a boxplot. For boxplot, the thick line in the box is median and the box spans from Q1 (25th percentile) to Q3 (75th percentile); the whiskers extend to the most extreme observation within 1.5 times the interquartile range (IQR=Q3–Q1) from the nearest quartile; outliers are represented by circles beyond the whiskers. Source data are provided as a Source Data file.

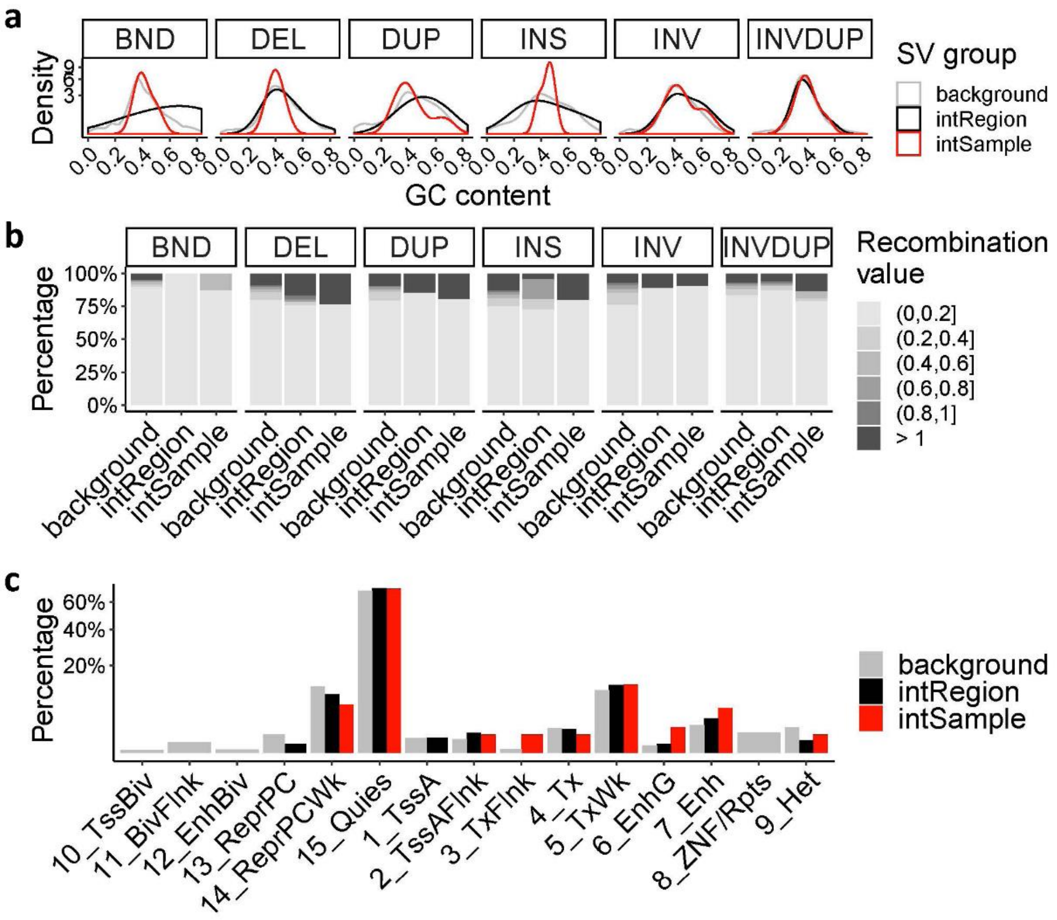

Figure S9. Genomic properties of structural variations in different regions. (**a**) Distribution of GC content of SVs in different regions per SV type. (**b**) Recombination value composition of SVs in different regions per SV type. (**c**) Chromatin state of SVs in different regions. Source data are provided as a Source Data file.

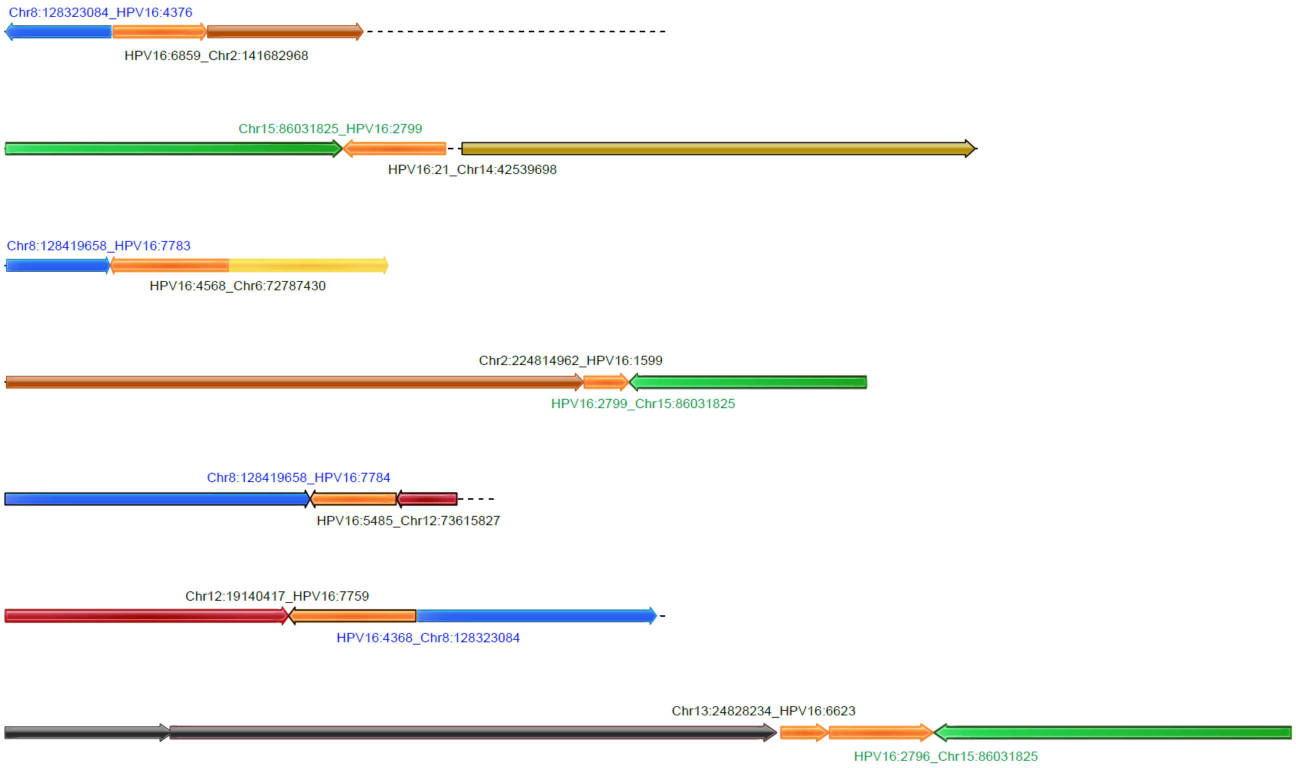

Figure S10. Seven additional clonal integration events with one supporting chimeric read in ZLR-11. Due to the inability to design PCR primers or the weak bands of PCR products, one of the breakpoints in these rare events has yet to be verified.

Uncropped scans of gels for Supplementary figure 1 and Supplementary figure 3:

Supplementary figure 1:

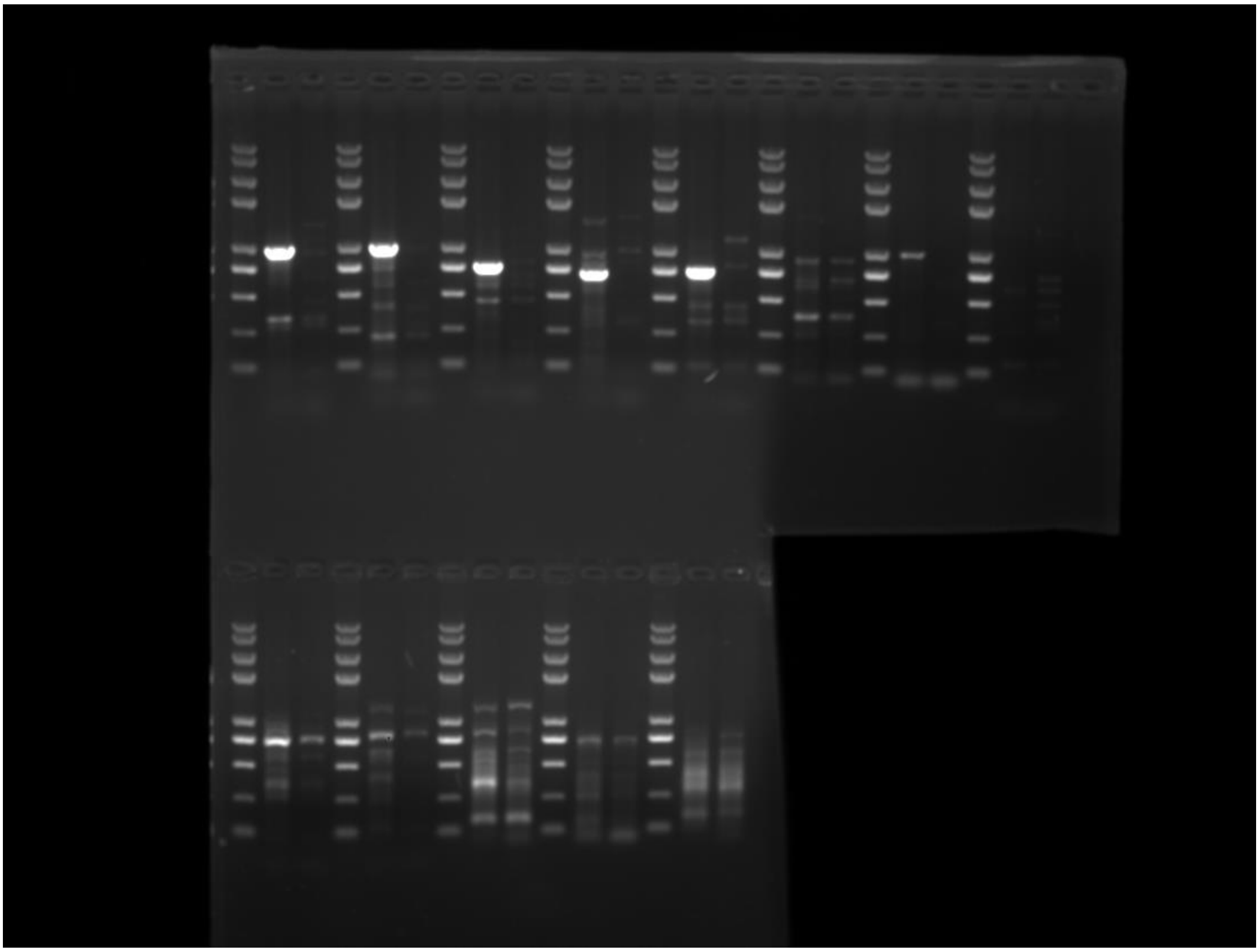

Supplementary figure 3:

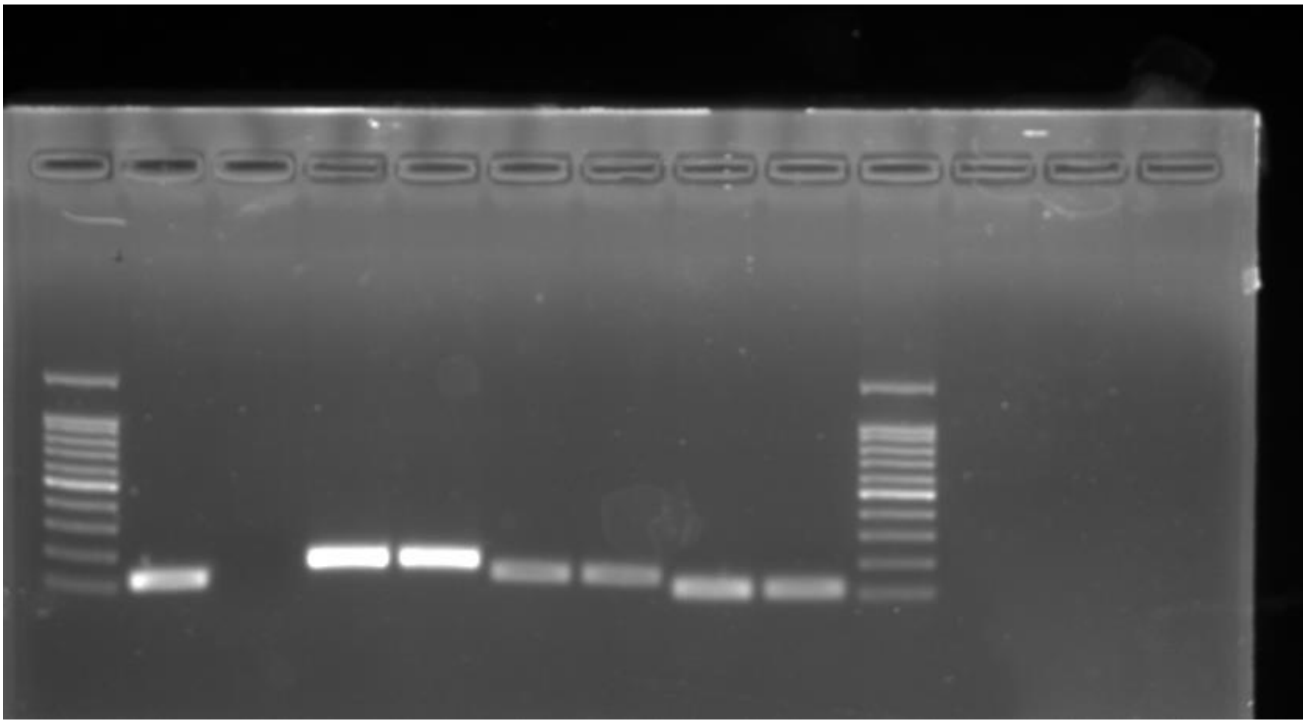

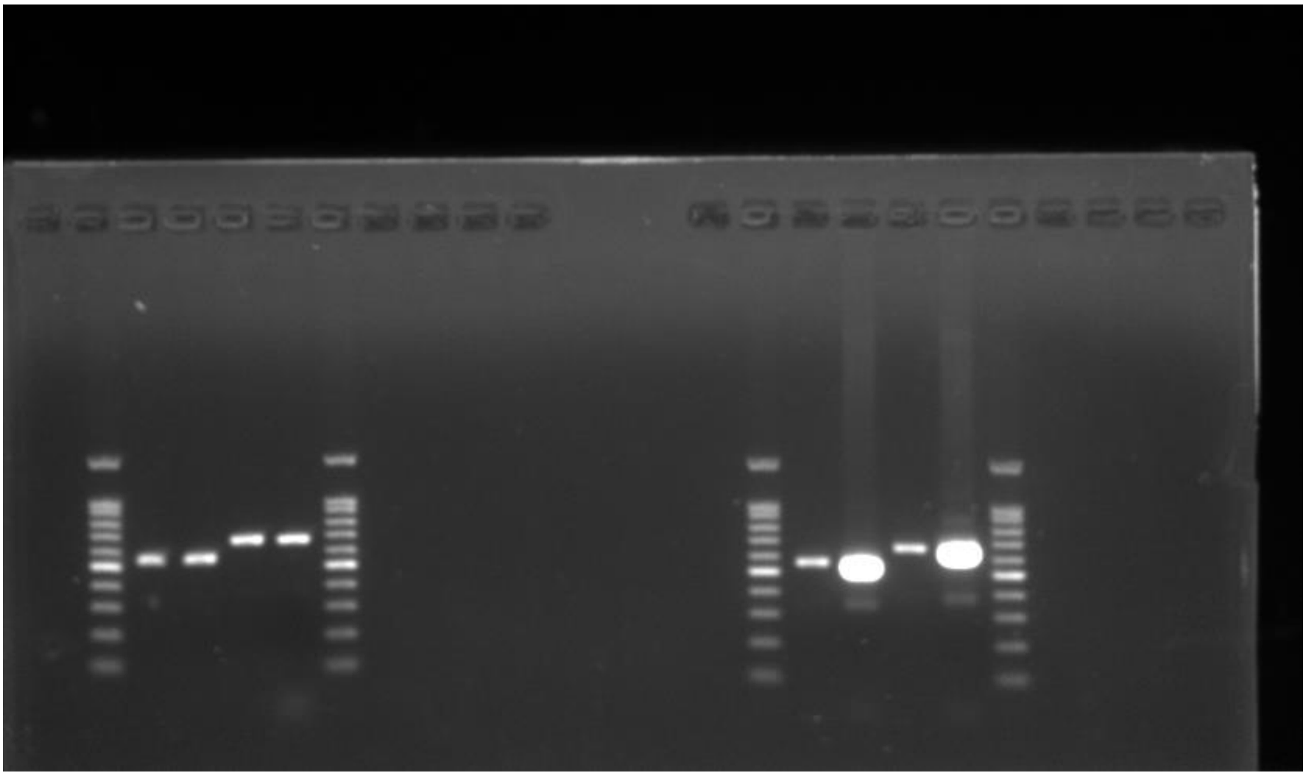

Supplement: Supplementary file 1 — Supplementary Information [file 41467_2022_30190_MOESM1_ESM.pdf]
